# Mercury Chloride Impacts on the Development of Erythrocytes and Megakaryocytes in Mice

**DOI:** 10.3390/toxics9100252

**Published:** 2021-10-07

**Authors:** Jinyi He, Yifan Zhao, Tingting Zhu, Peng Xue, Weiwei Zheng, Ye Yao, Weidong Qu, Xiaodong Jia, Rongzhu Lu, Miao He, Yubin Zhang

**Affiliations:** 1 School of Public Health and Key Laboratory of Public Health Safety, Ministry of Education, Fudan University, Shanghai 200032, China; 19211020037@fudan.edu.cn (J.H.); 19111020025@fudan.edu.cn (Y.Z.); 18211020029@fudan.edu.cn (T.Z.); pxue@fudan.edu.cn (P.X.); weiweizheng@fudan.edu.cn (W.Z.); yyao@fudan.edu.cn (Y.Y.); wdqu@fudan.edu.cn (W.Q.); 2 Shanghai Chemical Industry Park Medical Center, Shanghai 201507, China; jiaxiaodong@scipmc.com; 3 Department of Preventive Medicine and Public Health Laboratory Sciences, School of Medicine, Jiangsu University, Zhenjiang 212013, China; lurz@ujs.edu.cn; 4 State Key Laboratory of Medical Neurobiology, Institutes of Brain Science, Fudan University, Shanghai 200032, China; hem@fudan.edu.cn

**Keywords:** mercury chloride, erythro-megakaryopoiesis, erythropoietin receptor, Jak2/STAT5 signaling

## Abstract

Inorganic mercury (Hg^2+^) is a highly toxic heavy metal. The aim of this study was to investigate the impact of Hg^2+^ on the development of erythrocytes and megakaryocytes. B10.S mice (H-2^s^) and DBA/2 mice (H-2^d^) were administrated with 10 μM HgCl_2_ or 50 μM HgCl_2_ via drinking water for four weeks, and erythro-megakaryopoiesis was evaluated thereafter. The administration of 50 μM HgCl_2_ increased the number of erythrocytes and platelets in B10.S mice, which was not due to a reduced clearance for mature erythrocytes. The administration of 50 μM HgCl_2_, but not 10 μM HgCl_2_, increased the number of progenitors for erythrocytes and megakaryocytes in the bone marrow (BM) of B10.S mice, including erythroid-megakaryocyte progenitors (EMPs), burst-forming unit-erythroid progenitors (BFU-Es), colony-forming unit-erythroid progenitors (CFU-Es), and megakaryocyte progenitors (MkPs). Moreover, 50 μM HgCl_2_ caused EMPs to be more proliferative and possess an increased potential for differentiation into committed progenies in B10.S mice. Mechanistically, 50 μM HgCl_2_ increased the expression of the erythropoietin receptor (EPOR) in EMPs, thus enhancing the Jak2/STAT5 signaling pathway to promote erythro-megakaryopoiesis in B10.S mice. Conversely, 50 μM HgCl_2_ did not impact erythro-megakaryopoiesis in DBA/2 mice. This study may extend our current understanding for hematopoietic toxicology of Hg.

## 1. Introduction

Inorganic mercury (Hg^2+^) is a highly toxic heavy metal that broadly exists in human living environments. Hg^2+^ is derived from both the natural environment and human activities, the latter of which mainly include the combustion of fossil fuel and rubbish, mining activities, and sewage discharge; therefore, humans are exposed to Hg^2+^ typically through the inhalation of contaminated air or the intake of contaminated food or water [1,2,3].

Hg^2+^ has been shown to be toxic to erythrocytes, or red blood cells (RBC), which is likely due to a direct action of Hg^2+^ on erythrocytes. For instance, Hg chloride (HgCl_2_) increased the generation of reactive oxygen species (ROS) and reactive nitrogen species (RNS) to cause hemoglobin (HGB) oxidation in human erythrocytes in vitro [4]. HgCl_2_ suppressed the activation of the glucose 6-phosphate dehydrogenase (G6PD) enzyme in erythrocytes in rats both in vitro and in vivo [5]. In addition, an in vivo study reported that long-term treatment with HgCl_2_ on rats reduced the number of erythrocytes, caused lipid oxidation and chromosomal aberrations, and altered the activity of anti-oxidative enzymes [6]. Thus, Hg^2+^ significantly impairs the function of mature erythrocytes. So far, there have been no epidemiological studies to investigate the relationship between Hg^2+^ exposure and erythropoiesis in humans.

In mammals, all blood cells are differentiated from hematopoietic stem cells (HSCs), which are mainly localized in the bone marrow (BM) after birth [7]. Hematopoiesis refers to the generation of all blood cells from HSCs. HSCs are able to give rise to lymphoid cells, myeloid cells, and erythrocytes/megakaryocytes [8]. Erythro-megakaryopoiesis refers to the differentiation of erythrocytes and megakaryocytes/platelets, which primarily occurs in the BM after birth [8]. In the process of erythro-megakaryopoiesis, erythroid-megakaryocyte progenitors (EMPs) are capable of differentiating into both erythrocytes and megakaryocytes/platelets, with several intermediate steps including erythroid progenitors (EPs) and megakaryocyte progenitors (MkPs) [9]. EPs are comprised of two stages of progenitors, including the earlier burst-forming units-erythroid progenitors (BFU-Es) and the later colony-forming units-erythroid progenitors (CFU-Es) [10]. CFU-Es further differentiate into erythroblasts, which eventually lose their nuclei and become mature erythrocytes [10]. On the other hand, MkPs give rise to megakaryocytes and ultimately yield platelets [11]. Erythro-megakaryopoiesis is tightly regulated in vivo, and a variety of biological factors are well known to regulate this process [9]. For instance, erythropoietin (EPO) and granulocyte-macrophage colony-stimulating factor (GM-CSF) are able to promote erythropoiesis via the activation of the Janus kinase (Jak)2/signal transducer and activator of transcription (STAT)5 signaling pathway [12,13,14,15], while interferon (IFN) γ can lead to anemia partially through induction of the IFN regulatory factor (IRF)1/purine-rich nucleic acid binding protein.1 (PU.1) signaling pathway [16].

Environmental pollutants can significantly impact hematopoiesis [17,18]. We previously reported that heavy metals in the living environment such as cadmium, lead, and Hg significantly influenced the function of HSCs and the committed progenitors, leading to aberrant myelopoiesis and/or lymphopoiesis [19,20,21,22,23]. In terms of Hg, we found that Hg^2+^ impacted the proliferation of HSCs and critically relied on IFNγ-dependent on BM-resident macrophages (MΦ); Hg^2+^ increased the number of progenitors for granulocytes and MΦ in the BM of B10.S mice [19,24]. As Hg^2+^ is a toxicant in our living environment, understanding the impact of Hg^2+^ on the hematopoietic system is significant for public health.

Although it has been revealed that a direct action of Hg^2+^ on mature erythrocytes significantly impairs their function, to date, the impact of Hg^2+^ on the development of erythrocytes and megakaryocytes in the BM remains largely unknown. The aim of this study was to investigate the impact of Hg^2+^ on erythro-megakaryopoiesis. Hg^2+^-induced autoimmunity is critically dependent on H-2 haplotypes in mice [25]. Specifically, mice carrying the H-2^s^ haplotype are the most sensitive to Hg-induced autoimmunity, while mice carrying the H-2^b^ or H-2^d^ haplotype are relatively resistant to Hg-induced autoimmunity [25,26,27]. Although physiologically there is no inevitable connection between autoimmunity and erythro-megakaryopoiesis, Hg-induced autoimmunity has been shown to be critical for the occurrence of nephritis and encephalitis in mice during Hg exposure [28,29,30]. Thus, in the present study, we tested whether the Hg^2+^ impact on erythro-megakaryopoiesis was also related to H-2 haplotypes or autoimmunity in mice. We treated B10.S mice (H-2^s^), a strain sensitive to Hg^2+^-induced autoimmunity [28,31], and DBA/2 mice (H-2^d^), a strain resistant to Hg^2+^-induced autoimmunity [32,33], with HgCl_2_, and thereafter evaluated the impact of Hg^2+^ on erythro-megakaryopoiesis in the BM as well as the underlying mechanism. 

## 2. Materials and Methods

### 2.1. Mice and Hg Treatment

B10.S mice (H-2^s^) were obtained from the Jackson Laboratory (Bar Harbor, ME). DBA/2 mice (H-2^d^) were purchased from Shanghai SLAC Laboratory Animal Co. Ltd (Shanghai, China). Mice were housed in an animal facility with controlled temperature and humidity and an artificial 12-hour (hr) dark-light cycle at Fudan University. A total of five to six mice were housed in each cage with free access to food and drinking water. 

Adult mice aged from 6 to 8 weeks (w) were used, and both males and females were equally used. Mice were administrated with 10 μM (2 mg/L Hg) or 50 μM (10 mg/L Hg) HgCl_2_ (Sigma, St Louis, MO, USA) via drinking water for 4 w; regular drinking water was used as a vehicle control. The doses and time period of Hg treatment were according to our previous publications [19,28,29,34,35]. One criterion for dose selection was that the dose must be comparable to that of human exposure. The other criterion for dose selection was that the dose should be able to cause autoimmunity to test whether Hg impacts on erythro-megakaryopoiesis were related to autoimmunity. Our preliminary experiments suggested that the blood Hg concentration of mice treated with 10 μM or 50 μM HgCl_2_ was comparable to that of human subjects living in mining areas [36,37]. Our previous study indicated that 50 μM HgCl_2_ treatment was able to induce autoimmunity in mice carrying the H-2^s^ haplotype [28]. During the period of HgCl_2_ administration, no differences in water consumption were observed.

Assays performed on mice were conducted twice or thrice, and therefore the number of mice used for each individual experiment was not exactly the same. 

Fudan University Animal Care and Use Committee approved this work. All animal experiments complied with the ARRIVE guidelines and were carried out in accordance with the National Institutes of Health guide for the care and use of Laboratory animals, USA (NIH Publications No. 8023, revised 1978). 

### 2.2. Routine Blood Test (RBT)

Blood was harvested intracardially after anesthesia with carbon dioxide (CO_2_) and immediately placed in anti-clot tubes coated with ethylene diamine tetraacetic acid (EDTA). RBT was performed for fresh blood using an automatic blood cell analyzer, according to the instruction provided by the manufacturer (BC-2800vet, Shenzhen, China). 

### 2.3. Hg Measurement

Blood was harvested with anti-clot tubes as described above. BM homogenate was harvested as we have previously reported [19,20,38,39]. Briefly, BM from each leg was flushed out with 200 μL of a homogenate buffer containing IGEPAL CA-630, Tris-Cl, NaCl, EDTA, sodium (Na)-orthovanadate, Na fluoride (NaF), and proteinase inhibitors (Sigma-Aldrich, St Louis, MO, USA). A bicinchoninic acid (BCA) kit (Pierce, Rockford, IL, USA) was used to quantify the total protein concentration of the homogenate, and the protocol was according to the manufacturer’s instructions. Total Hg was measured using atomic fluorescence spectrometry (Agilent, Santa Clara, CA, USA), as others have previously reported [40,41]. Hg concentration in the blood and BM homogenate was reported as μg/L and μg/kg protein (part per billion, ppb), respectively. 

### 2.4. Antibodies (Ab) and Flow Cytometry

Ab (clone) and fluorescein used to stain progenitors, erythroblasts and leukocytes included lineage cocktail (Lin, CD3 (145-2C11), CD11b (M1/70), B220 (RA3-6B2), Gr-1 (RB6-8C5) and Ter119 (TER-119))-FITC or biotin, c-Kit-APC (2B8), Scal-1-PerCP-Cy5.5 (D7), CD150-PE-Cy7 (TC15-12F12.2), CD150-APC (TC15-12F12.2), CD41-FITC (MWReg30), CD41-PE (MWReg30), CD105-PB (MJ7/18); CD105-PE-Cy7 (MJ7/18), CD71-PE-Cy7 (RI7217), CD45-PE-Cy7 (30-F11), CD11b-PerCP-Cy5.5 (M1/70), Ly6G-PE (1/A8), F4/80-PE-Cy7 (BM8), Ki67-PE (16A8), p-STAT1-PE (pY701); p-STAT3-PE (Tyr705), streptavidin (SA)-PB, SA-APC-Cy7, anti-rabbit Ab-PE, anti-rabbit Ab-FITC and anti-rabbit Ab-APC (Biolegend, San Diego, CA, USA), and rabbit anti-mouse p-Jak1 (Tyr1022) (Nanjing Jiancheng, China), rabbit anti-mouse p-Jak2 (Tyr1007/1008), rabbit anti-mouse p-STAT5 (Tyr694), and rabbit anti-mouse EPO receptor (EPOR) (Beyotime Biotechnology, China), and rat anti-mouse CD16/32 (Fc block, 2.4G2) (BD Biosciences, San Diego, CA), and 4’6-diamidino-2-phenylindole (DAPI) (Sigma, St Louis, MO, USA). 

Mature erythrocytes were lysed from splenic and BM cells using an ammonia-based lysis buffer. Live cells were incubated with Fc block on ice for 20 min, and surface staining was performed thereafter by incubating cells with fluorescein-conjugated Ab on ice in the dark for 30 min. Nuclear staining for transcription factors was performed after surface staining using a kit from BD Biosciences; the protocol was according to the instructions provided by the manufacturer. The surface markers used for identifying numerous erythroid and megakaryocyte progenitors as well as erythroblasts are summarized in Table 1, according to previously published literature [42,43,44,45,46]. The stained cells were analyzed through a BD LSRFortessa instrument. 

### 2.5. RBC Clearance Assay

Fresh blood harvested from B10.S mice was placed in EDTA-coated anti-clot tubes and then incubated with sulfo-NHS-LC-biotin Na (0.2 mg/ml; MedChemExpress, Monmouth Junction, NJ, USA) at 37 °C for 30 min to label with biotin, according to a previous report [16]. After washing with phosphate buffer saline (PBS), 200 μL of biotin-labeled blood was intravenously injected into B10.S mice that had been treated with 50 μM HgCl_2_ or control for 4 w. The recipient mice were sacrificed to analyze the biotin^+^ RBCs in the blood and biotin^+^ MΦ in the spleen three days after the blood transfer; HgCl_2_ or control treatment was continuously provided during this period.

### 2.6. CFU Assay

A total of 2 × 10^4^ (2e^4^) BM cells that had been lysed with RBCs were placed in each 35 mm tissue culture Petri-dish containing 1mL of a semi-liquid methocellulose medium (MethoCult, GF M3434, Stem Cell Technologies, Vancouver, BC, Canada). The dishes were incubated at 37 °C and 5% CO_2_ with saturated humidity for 6 d, and the number of CFU-Es was quantified thereafter under a microscope, as per a protocol we have previously reported [24,38].

### 2.7. Hematoxylin-Eosin (H&E) Staining

Legs were harvested from mice treated with 50 μM HgCl_2_ or the control for 4 w, and the legs were thereafter fixed with 4% paraformaldehyde (PFA) for 24 h. The legs were then decalcified, embeded with paraffin, and sliced. After that, the sections were coated on charged slides and regular H&E staining was performed. 

### 2.8. EMPs Intervention and Differentiation Assay In Vitro

EMPs were purified from the BM of B10.S mice treated with 50 μM HgCl_2_ or the control through a fluorescence-activated cell sorting (FACS) technique using a BD Aria II instrument, according to their surface markers listed in Table 1. A total of 500 EMPs were placed in each well of 96-well plates with 200 μL of an erythro-megakaryocyte differentiation medium (StemSpan™ SFEM, STEMCELL Technologies, BC, Canada) supplemented with interleukin-3 (IL-3, 10 ng/mL) and stem cell factor (SCF, 100 ng/mL) (Biolegend, San Diego, CA, USA), and in the presence or absence of Jak2 inhibitor fedratinib (3 nM), STAT5 inhibitor STAT5-IN-1 (50 μM) (MedChemExpress, Monmouth Junction, NJ, USA), or EPO (100 ng/ mL) (Novoprotein, Shanghai, China). After incubation at 37 °C and 5% CO_2_ for 16 h, the number of BFU-Es, CFU-Es, and MkPs, and the activation of the Jak2/STAT5 signaling pathway were analyzed through a BD LSRFortessa flow cytometry instrument.

A total of 500 FACS-purified EMPs from the BM of control B10.S mice were placed in each well of 96-well plates with 200 μL of an erythro-megakaryocyte differentiation medium as described above in the presence or absence of HgCl_2_ (200 μg/L) and/or EPO (100 ng/mL). After incubation at 37 °C and 5% CO_2_ for 16 h, the number of BFU-Es, CFU-Es, and MkPs and the activation of the Jak2/STAT5 signaling pathway were measured.

### 2.9. Real-Time Quantitative Polymerase Chain Reaction (q-PCR)

RNA was extracted from the kidney of B10.S mice as well as FACS-purified EMP from the BM of B10.S mice. The extracted RNA was reversely transcribed into cDNA. The q-PCR with SYBR green was used to quantify the expression of mRNA for cyclin-dependent kinase (CDK)2, CDK4, EPO, and EPOR using kits and an instrument purchased from Thermo Fisher Scientific (Walsham, MA); the expression of β-actin mRNA was used as an internal control. The results reported as the fold change relative to the control were calculated by the ΔΔCT method. The information for forward and reverse primers for target genes was summarized in Table 2. 

### 2.10. Confocol Imaging

The procedeure for pareparing the coated sections of legs from mice treated with 50 μM HgCl_2_ or the control was the same as that used for H&E staining. After that, the sections were hydrated, and an antigen retrieval procedure was then performed. After incubation with goat serum, the sections were incubated in the presence of lineage cocktail (CD3 (145-2C11), CD11b (M1/70), B220 (RA3-6B2), Gr-1 (RB6-8C5) and Ter119 (TER-119))-Alexa Fluor 488, Ki67-APC (16A8), EPOR-PE, and DAPI. The stained sections were analyzed with a confocal microscope (Nikon, Japan).

### 2.11. Enzyme-Linked Immunosorbent Assay (ELISA)

ELISA kits were purchased from Biolegend to quantify the concentration of EPO in serum and BM homogenate, and protocol was according to the manufacturer’s instructions. 

### 2.12. Statistics

Data were presented as mean or mean ± standard deviation (StD). Data comparisons between the two groups were analyzed with Student’s *t*-tests. Data comparisons among multiple groups were first analyzed with a one-way analysis of variance (ANOVA), and if there were significant differences, data were further analyzed using Student-Newman-Keuls tests to compare between the two groups. The value *p* < 0.05 was considered as the level of a significant difference.

## 3. Results

### 3.1. Hg Concentration in the Blood and BM

To evaluate the level of Hg burden in mice, we measured total Hg concentration in the blood and BM using atomic fluorescence spectrometry. The concentration of Hg in the blood of B10.S mice treated with 10 μM or 50 μM HgCl_2_ was 26.5 μg/L (ppb) and 242.5 μg/L (ppb), respectively; the concentration of Hg in the blood of DBA/2 mice treated with 50 μM HgCl_2_ was 487.2 μg/L (ppb) (Table 3). Although Hg was not detectable in the BM of B10.S mice treated with 10 μM HgCl_2_, Hg was detectable in the BM of mice treated with 50 μM HgCl_2_; the level of Hg in the BM of B10.S mice and DBA/2 mice treated with 50 μM HgCl_2_ was 2500.0 μg/kg protein (ppb) and 2200.0 μg/kg protein (ppb), respectively (Table 3). 

### 3.2. HgCl_2_ Increases the Number of Mature RBCs and Platelets in the Blood of B10.S Mice

**Figure 1:**
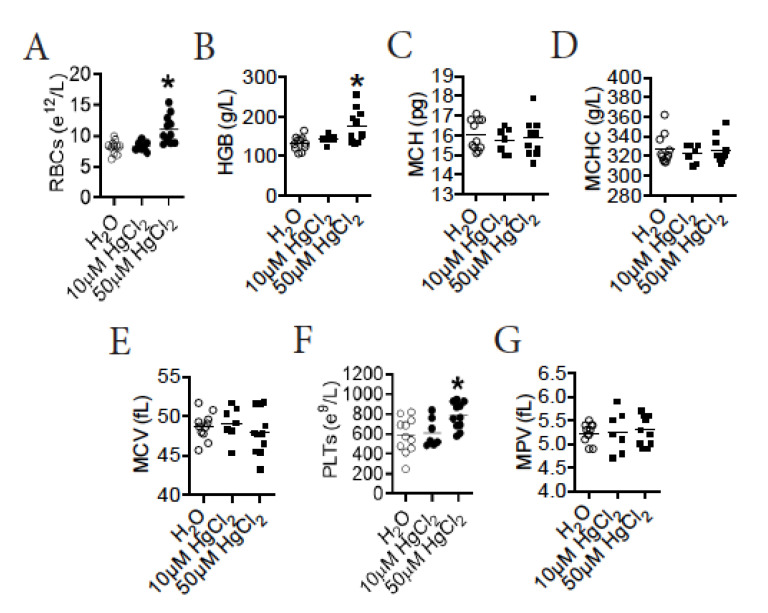
HgCl_2_ increases the number of erythrocytes and platelets in the blood of B10.S mice. B10.S mice were treated with 10 μM or 50 μM HgCl_2_ for 4 w, and RBT was performed thereafter. (**A**) The concentration of RBCs. (**B**) The concentration of HGB. (**C**) The MCH. (**D**) The MCH concentration (MCHC). (**E**) The MCV. (**F**) The concentration of platelets (PLTs). (**G**) The MPV. Each dot represents one mouse, and a total of 7 to 11 mice were used for each group. Asterisk indicates a significant difference compared to the counterpart control group. *p* < 0.05 was considered as the level of significant difference.

To test the impact of HgCl_2_ on erythro-megakaryopoiesis, we performed RBT on B10.S mice after exposure to HgCl_2_. We found that treatment with 50 μM HgCl_2_, but not 10 μM HgCl_2_, increased the number of mature RBCs in the blood of B10.S mice (Figure 1A). Accordingly, treatment with 50 μM HgCl_2_, but not 10 μM HgCl_2_, also increased the concentration of hemoglobin (HGB) in the blood of B10.S mice (Figure 1B). HgCl_2_ treatment did not influence the mean corpuscular hemoglobin (MCH), the MCH concentration (MCHC), or the mean corpuscular volume (MCV) in the blood of B10.S mice (Figure 1C–E). Consistently, treatment with 50 μM HgCl_2_, but not 10 μM HgCl_2_, increased the number of platelets (PLTs), but not the mean platelet volume (MPV) in the blood of B10.S mice (Figure 1F,G). Therefore, treatment with 50 μM HgCl_2_ increased the number of mature RBCs and platelets in the blood of B10.S mice.

### 3.3. HgCl_2_ Does Not Impact the Clearance of Mature RBCs in B10.S Mice

Mature RBCs are mainly cleared by MΦ in mice [16,47]. As treatment with 50 μM HgCl_2_ increased the number of mature RBCs in the blood of B10.S mice, we sought to test whether the increased number of mature RBCs by HgCl_2_ exposure was caused by repressed clearance. To test this hypothesis, biotinylated mature RBCs were transferred into 50 μM HgCl_2_-treated B10.S mice or control B10.S mice through intravenous injection, and the clearance of the donated RBCs was measured thereafter (Supplementary Figure S1A). We found that B10.S mice treated with 50 μM HgCl_2_ or the control had comparable residual biotinylated RBCs in their blood (Supplementary Figure S1B,C). Consistent with this, B10.S mice treated with 50 μM HgCl_2_ or the control had a similar number of splenic MΦ (gated on CD11b^+^ly6G^-^F4/80^+^) devouring the donor RBCs (biotin^+^ MΦ) (Supplementary Figure S1D). Thus, we concluded that the increased number of mature RBCs by 50 μM HgCl_2_ treatment was not due to a reduced clearance of RBCs in B10.S mice.

### 3.4. HgCl_2_ Increases the Development of Erythrocytes and Megakaryocytes/Platelets in the BM of B10.S Mice

**Figure 2:**
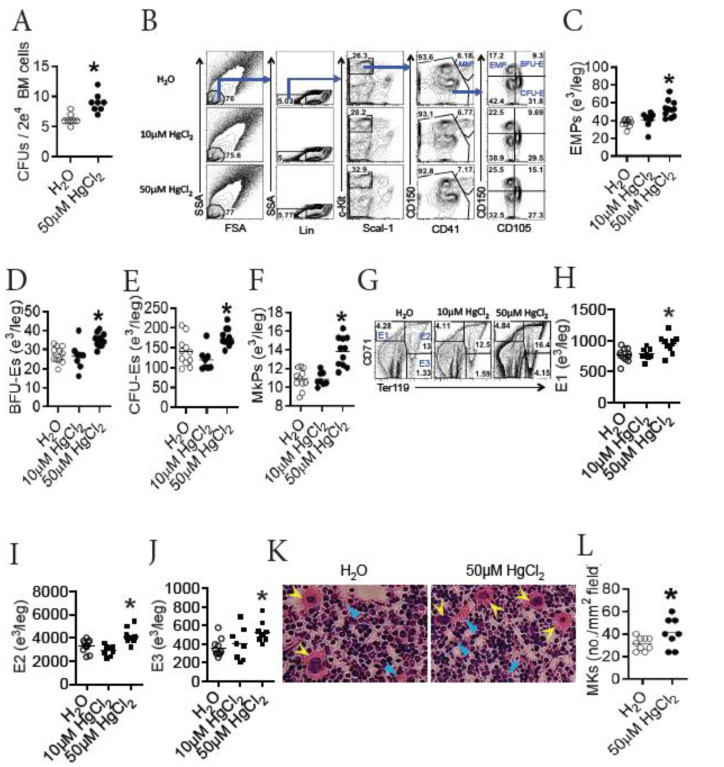
HgCl_2_ increases the development of erythrocytes and megakaryocytes/platelets in the BM of B10.S mice. B10.S mice were treated with 10 μM or 50 μM HgCl_2_ for 4 w, and the potential of BM cells to generate CFU-Es as well as erythro-megakaryopoiesis in the BM were measured thereafter. (**A**) The number of CFU-Es derived from BM cells ex vivo. (**B**) Representative flow plots for EMPs, BFU-Es, CFU-Es and MkPs in the BM. (**C**) Quantification of EMPs in the BM as indicated in (**B**). (**D**) Quantification of BFU-Es in the BM as indicated in (**B**). (**E**) Quantification of CFU-Es in the BM as indicated in (**B**). (**F**) Quantification of MkPs in the BM as indicated in (**B**). (**G**) Representative flow plots for erythroblasts (E1, E2, and E3) in the BM. (**H**) Quantification of E1 in the BM as indicated in (**G**). (**I**) Quantification of E2 in the BM as indicated in (**G**). (**J**) Quantification of E3 in the BM as indicated in (**G**). (**K**) Representative histological images for H&E staining of BM sections from B10.S mice treated with 50 μM HgCl_2_ or the control. Yellow arrows and blue arrows point to megakaryocytes and RBCs, respectively. (**L**) Quantification of MK for each mm^2^ of the BM field as indicated in K. Each dot represents one mouse, and a total of 8 to 11 mice were used for each group. Asterisk indicates a significant difference compared to the counterpart control group. *p* < 0.05 was considered as the level of significant difference.

Erythro-megakaryopoiesis primarily occurs in the BM after birth in mammals [8]. As HgCl_2_ did not reduce the clearance of mature RBCs in B10.S mice, we sought to determine whether HgCl_2_ promoted the generation of erythrocytes and megakaryocytes in the BM of B10.S mice. To test this, we evaluated the potential of BM cells to generate CFU-Es. We found that treatment with 50 μM HgCl_2_ increased the number of CFU-Es derived from the BM cells of B10.S mice ex vivo (Figure 2A), indicating that HgCl_2_ drove erythropoiesis in the BM of B10.S mice. During erythro-megakaryopoiesis, EMPs differentiate into both erythrocyte and megakaryocyte lineages. Chronologically, the erythrocyte lineage includes BFU-Es (also known as pre-CFU-Es), CFU-Es, erythroblast 1 (E1), E2, E3, and mature RBCs, and the megakaryocyte lineage includes MkPs, megakaryocytes, and platelets, as schematized in Supplementary Figure S2A. We further measured the number of different types of progenitors for erythrocytes and megakaryocytes in the BM of B10.S mice after exposure to HgCl_2_. We found that treatment with 50 μM HgCl_2_, but not 10 μM HgCl_2_, increased the number of progenitors for erythrocytes, including EMPs, BFU-Es, and CFU-Es in the BM of B10.S mice (Figure 2B–E). In addition, treatment with 50 μM HgCl_2_, but not 10 μM HgCl_2_, also increased the number of MkPs in the BM of B10.S mice (Figure 2B,F).

At the late stage of erythropoiesis, CFU-Es differentiate into erythroblasts (here refers to E1, E2, and E3, according to their surface expression of CD71 and Ter119 as summarized in Table 1) and eventually become mature RBCs, as schematized in Supplementary Figure S2A. We measured the number of erythroblasts in the BM of B10.S mice after exposure to HgCl_2_. As expected, treatment with 50 μM HgCl_2_, but not 10 μM HgCl_2_, increased the number of E1, E2, and E3 in the BM of B10.S mice (Figure 2G–J). In addition, we performed histological staining for the BM of B10.S mice treated with 50 μM HgCl_2_ or the control, and we observed morphologically that treatment with 50 μM HgCl_2_ increased the number of megakaryocytes as well as mature RBCs in the BM (Figure 2K–L).

In mouse models, increased erythropoiesis in the BM was associated with suppressed erythropoiesis in the periphery, e.g., the spleen, and vice versa [16]. As treatment with HgCl_2_ drove erythro-megakaryopoiesis in the BM, we sought to determine whether HgCl_2_ suppressed erythropoiesis in the spleen of B10.S mice. As inferred, in contrast to the increased erythropoiesis in the BM by treatment with 50 μM HgCl_2_, the number of E1, E2 and E3 in the spleen was indeed reduced in B10.S mice after treatment with 50 μM HgCl_2_, but not treatment with 10 μM HgCl_2_ (Supplementary Figure S3A–D).

We therefore concluded that treatment with 50 μM HgCl_2_ promoted the development of erythrocytes and megakaryocytes/platelets in the BM of B10.S mice.

### 3.5. HgCl_2_ Drives the Proliferation and Differentiation of EMPs in the BM of B10.S Mice

**Figure 3:**
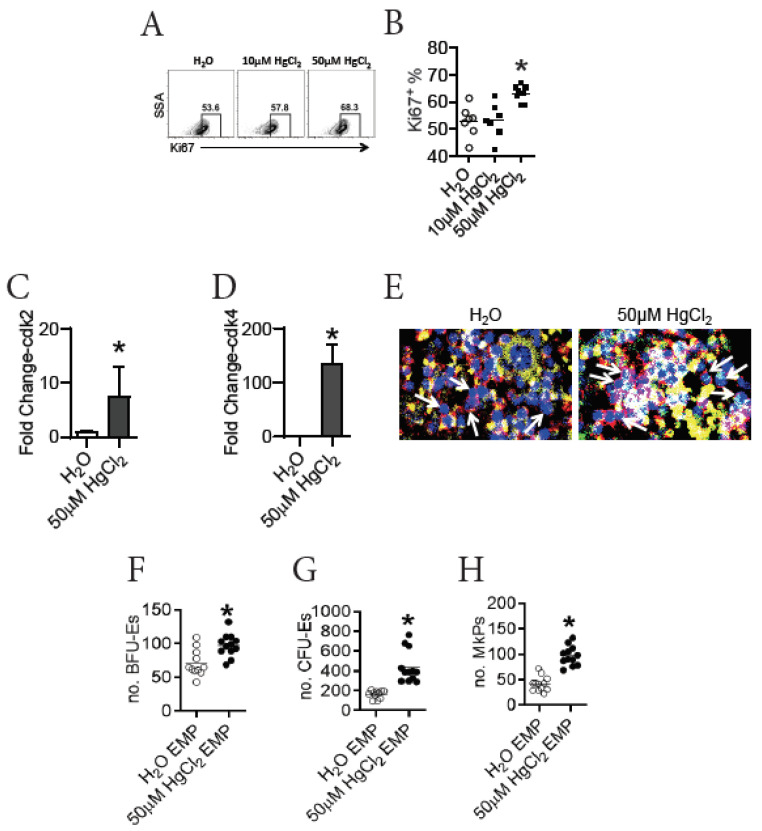
HgCl_2_ increases the proliferation and differentiation of EMPs in the BM of B10.S mice. B10.S mice were treated with 10 μM or 50 μM HgCl_2_ via drinking water for 4 w, and the proliferation of EMPs in the BM were measured. EMPs were purified from the BM of B10.S mice treated with the control or 50 μM HgCl_2_ through FACS sorting, and the EMPs were measured for the expression of cdk2 and cdk4 mRNA thereafter; the EMPs were also cultured in vitro for 16 h to evaluate their potential for differentiation into BFU-Es, CFU-Es, and MkPs. (**A**) Representative flow plots for the expression of Ki67 in EMPs in the BM. (**B**) Quantification of the expression of Ki67 in the EMPs as indicated in (**A**). (**C**) The expression of cdk2 mRNA (fold change) in EMPs in the BM. (**D**) The expression of cdk4 mRNA (fold change) in EMPs in the BM. (**E**) Representative confocal images for the proliferation (Ki67^+^) of erythrocyte progenitors as indicated by Lin^-^EPOR^+^DAPI^+^ cells in the BM of B10.S mice treated with 50 μM HgCl_2_ or the control (yellow for Lin, red for EPOR, green for Ki67, and blue for DAPI). (**F**) The number of BFU-Es in the differentiation assay. (**G**) The number of CFU-Es in the differentiation assay. (**H**) The number of MkPs in the differentiation assay. Each dot represents one mouse or one sample, and a total of 7 to 12 mice or samples were used for each group. Asterisk indicates a significant difference compared to the counterpart control group. *p* < 0.05 was considered as the level of significant difference.

EMPs are recognized as the ancestors for both erythrocytes and megakaryocytes/platelets. As HgCl_2_ promoted erythro-megakaryopoiesis in the BM of B10.S mice, we sought to determine whether HgCl_2_ increased the potential for EMP differentiation in B10.S mice. To test this, we first evaluated the proliferation of EMPs in the BM of B10.S mice after exposure to HgCl_2_. We found that treatment with 50 μM HgCl_2_, but not 10 μM HgCl_2_, increased the expression of Ki67 in EMPs in the BM of B10.S mice (Figure 3A,B), indicating that treatment with 50 μM HgCl_2_ increased the proliferation of EMPs. In accordance with this, treatment with 50 μM HgCl_2_ increased the expression of mRNA for CDK2 and CDK4, which are kinases driving cell cycling [48,49], in FACS-purified EMPs from the BM of B10.S mice (Figure 3C,D). Moreover, a confocal imaging assay revealed that treatment with 50 μM HgCl_2_ increased the expression of Ki67 in Lin^-^EPOR^+^ cells in the BM of B10.S mice (Figure 3E), further supporting the inference that 50 μM HgCl_2_ drove the proliferation of erythrocyte lineage progenitors in B10.S mice.

Next, we directly tested the potential for EMPs to differentiation using an ex vivo assay. FACS-purified EMPs from the BM of B10.S mice treated with 50 μM HgCl_2_ or the control for 4 w were placed in a differentiation medium to evaluate their potential for generating progenitors for erythrocytes and megakaryocytes (Supplementary Figure S4A). As surmised, FACS-purified EMPs from B10.S mice treated with 50 μM HgCl_2_ gave rise to more BFU-Es, CFU-Es, and MkPs than FACS-purified EMPs from the control B10.S mice did (Figure 3F–H), indicating that treatment with 50 μM HgCl_2_ increased the potential for EMPs to give rise to more progenies in the BM of B10.S mice.

Taken together, we concluded that treatment with 50 μM HgCl_2_ drove the proliferation and differentiation of EMPs in the BM of B10.S mice.

### 3.6. HgCl_2_ Activates the Jak2/STAT5 Signaling Pathway to Promote EMP Differentiation in the BM of B10.S Mice

**Figure 4:**
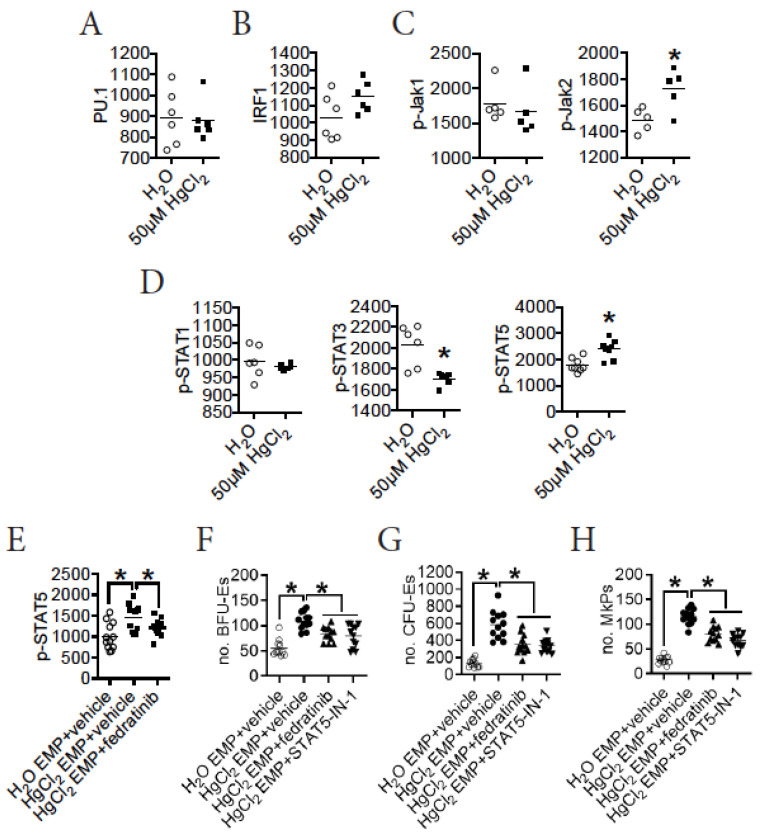
HgCl_2_ activates the Jak2/STAT5 pathway to promote the differentiation of EMPs in B10.S mice. B10.S mice were treated with the control or 50 μM HgCl_2_ for 4 w, and multiple signaling molecules involved in erythropoiesis including PU.1, IRF1, p-Jak1, p-Jak2, p-STAT1, p-STAT3, and p-STAT5 were measured for EMPs in the BM thereafter; FACS-purified EMPs from the BM were treated in the presence or absence of inhibitors for the Jak2/STAT5 signaling pathway in vitro for 16 h to evaluate its impact on STAT5 signaling and EMP differentiation into BFU-Es, CFU-Es, and MkPs. (**A**) The expression of PU.1 (mean fluoresce intensity, MFI) in EMPs in the BM. (**B**) The expression of IRF1 (MFI) in EMPs in the BM. (**C**) The expression of p-Jak1 and p-Jak2 (MFI) in EMPs in the BM. (**D**) The expression of p-STAT1, p-STAT3, and p-STAT5 (MFI) in EMPs in the BM. (**E**) The expression of p-STAT5 (MFI) in EMPs in the presence or absence of Jak2 inhibitor (fedratinib) in vitro. (**F**) The number of BFU-Es in the in vitro assay. (**G**) The number of CFU-Es in the in vitro assay. (**H**) The number of MkPs in the in vitro assay. Each dot represents one mouse or one sample, and a total of 5 to 12 mice were used for each group. Asterisk indicates a significant difference compared to the counterpart control group. *p* < 0.05 was considered as the level of significant difference.

To investigate the signaling pathway involved in erythro-megakaryopoiesis that was impacted by HgCl_2_ exposure, we analyzed numerous signal molecules and transcription factors that were known to impact erythropoiesis in the BM of B10.S mice. We measured the expression of PU.1 and IRF1, two nuclear transcription factors that were reported to suppress erythropoiesis in mice [16]. We found that treatment with 50 μM HgCl_2_ did not affect the expression of PU.1 or IRF1 in EMPs in the BM of B10.S mice (Figure 4A,B). The Jak/STAT signaling pathways have been recognized as crucial for erythropoiesis [12]. We therefore measured the activation of Jak/STAT signaling pathways in EMPs in the BM of B10.S mice after exposure to HgCl_2_. We found that treatment with 50 μM HgCl_2_ increased the expression of p-Jak2, but not p-Jak1, in the EMPs in the BM of B10.S mice (Figure 4C). Interestingly, while treatment with 50 μM HgCl_2_ did not influence the expression of p-STAT1, treatment with HgCl_2_ reduced the expression of p-STAT3 and increased the expression of p-STAT5 in the EMPs in the BM of B10.S mice (Figure 4D). 

Activation of the Jak2/STAT5 signaling pathway has been suggested to be crucial in driving erythropoiesis in mice [12,13]. To test whether HgCl_2_ promoted erythropoiesis via activation of this pathway, we used an ex vivo assay to test the role of its activation in EMPs in erythro-megakaryopoiesis in the BM of B10.S mice during HgCl_2_ exposure (Supplementary Figure S4B). We confirmed that the increased expression of p-STAT5 in EMPs was mediated by the activation of Jak2 in EMPs in the BM of B10.S mice during HgCl_2_ exposure, as the increased expression of p-STAT5 in EMPs purified from the BM of HgCl_2_-treated B10.S mice was diminished in the presence of the Jak2 specific inhibitor fedratinib (Figure 4E). In addition, we found that the increased potential for FACS-purified EMPs from the BM of B10.S mice treated with 50 μM HgCl_2_ to give rise to BFU-Es, CFU-Es, and MkPs was recovered in the presence of the Jak2-specific inhibitor fedratinib or the STAT5-specific inhibitor STAT5-IN-1 ex vivo (Figure 4F–H). 

Taken together, these observations suggested that treatment with 50 μM HgCl_2_ drove erythro-megakaryopoiesis via enhanced activation of the Jak2/STAT5 signaling pathway in the BM of B10.S mice.

### 3.7. A Direct Action of HgCl_2_ on EMPs Suppresses Their Differentiation in the BM of B10.S Mice

**Figure 5:**
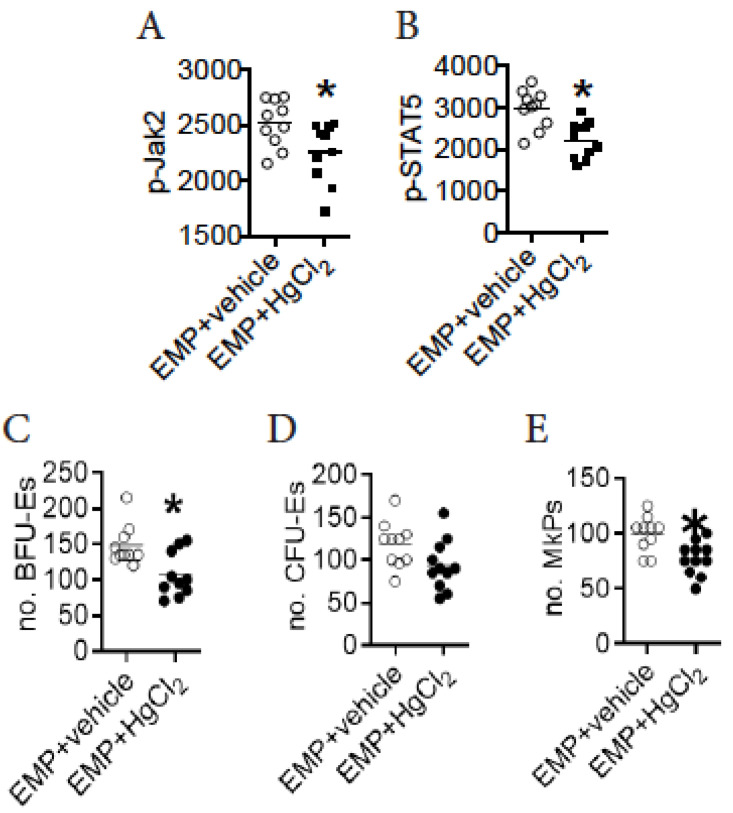
A direct action of HgCl_2_ on EMPs suppresses their differentiation in the BM of B10.S mice. FACS-purified EMPs from the BM of B10.S mice were treated with HgCl_2_ or the vehicle in vitro for 16 h and the Jak2/STAT5 signaling pathway and differentiation of EMPs into BFU-Es, CFU-Es, and MkPs were measured thereafter. (**A**) The expression of p-Jak2 (MFI) in EMPs. (**B**) The expression of p-STAT5 (MFI) in EMPs. (**C**) The number of BFU-Es in the differentiation **assay**. (**D**) The number of CFU-Es in the differentiation assay. (**E**) The number of MkPs in the differentiation assay. Each dot represents one mouse, and a total of 10 mice or samples were used for each group. Asterisk indicates a significant difference compared to the counterpart control group. *p* < 0.05 was considered as the level of significant difference.

As activation of the Jak2/STAT5 signaling pathway drove the differentiation of EMPs during HgCl_2_ exposure, we sought to determine whether the activation of this pathway was caused by a direct action of HgCl_2_ on EMPs. To test this, FACS-purified EMPs from the BM of B10.S mice were treated with HgCl_2_ in vitro to evaluate the activation of this pathway as well as their differentiation potential (Supplementary Figure S4C). Intriguingly, treatment with HgCl_2_ on EMPs in vitro reduced their expression of p-Jak2 and p-STAT5 (Figure 5A,B), indicating that a direct action of HgCl_2_ suppressed the activation of the pathway in EMPs. In line with this, treatment with HgCl_2_ on EMPs in vitro suppressed the potential for EMP differentiation, since FACS-purified EMPs from the BM of B10.S mice tended to give rise to fewer BFU-Es, CFU-Es, and MkPs in the presence of HgCl_2_ in vitro (Figure 5C–E). These observations indicated that a direct action of HgCl_2_ on EMPs suppressed their potential for differentiation in the BM of B10.S mice. 

### 3.8. HgCl_2_ Does Not Impact EPO Production, but Increases the Expression of EPOR to Enhance the Jak2/STAT5 Signaling Pathway in EMPs in the BM of B10.S Mice 

**Figure 6:**
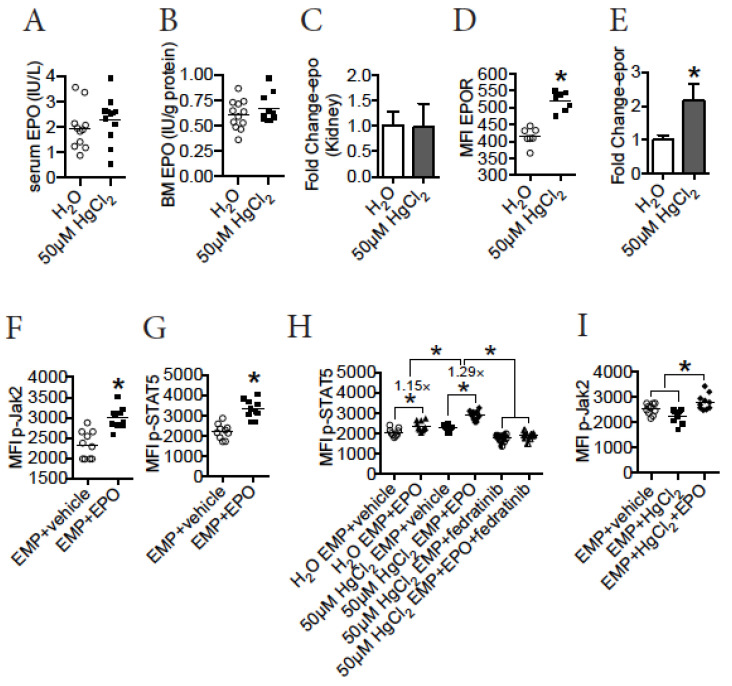
HgCl_2_ does not impact the production of EPO, but increases the expression of EPOR in EMPs in B10.S mice. EPO protein in the serum and BM, EPO mRNA in the kidney, and EPOR in EMPs in the BM in B10.S mice treated with 50 μM HgCl_2_ or the control for 4 w were measured. FACS-purified EMPs from the BM of B10.S mice were treated with HgCl_2_ or vehicle or EPO in vitro, and the Jak2/STAT5 signaling pathway and differentiation of EMPs were measured thereafter. (**A**) EPO protein (IU/L) in the serum of B10.S mice treated with 50 μM HgCl_2_ or water for 4 w. (**B**) EPO protein (IU/g protein) in the BM of B10.S mice treated with 50 μM HgCl_2_ or water for 4 w. (**C**) EPO mRNA (fold change) in the kidney of B10.S mice treated with 50 μM HgCl_2_ or water for 4 w. (**D**) The expression of EPOR (MFI) in EMPs in the BM of B10.S mice treated with 50 μM HgCl_2_ or water for 4 w. (**E**) The expression of EPOR mRNA (fold change) in FACS-purified EMPs from the BM of B10.S mice treated with 50 μM HgCl_2_ or water for 4 w. (**F**) EMPs were purified from the BM of control B10.S mice, and the FACS-purified EMPs were treated thereafter with EPO or vehicle in vitro for 16 h. After that, the expression of p-Jak2 (MFI) in EMPs was measured. (**G**) EMPs were purified from the BM of control B10.S mice, and the FACS-purified EMPs were treated thereafter with EPO or vehicle in vitro for 16 h. After that, the expression of p-STAT5 (MFI) in EMPs was measured. (**H**) EMPs were purified from the BM of B10.S mice treated with 50μM HgCl_2_ or water for 4 w, and the EMPs were treated thereafter with EPO or vehicle in the presence or absence of the Jak2 inhibitor fedratinib for 16 h in vitro. After that, the expression of p-STAT5 (MFI) in the EMPs was measured. (**I**) EMPs were purified from the BM of control B10.S mice, and the FACS-purified EMPs were treated thereafter with HgCl_2_ and/or EPO or vehicle in vitro for 16 h. After that, the expression of p-Jak2 (MFI) in EMPs was measured. Each dot represents one mouse or one sample, and a total of 5 to 12 mice or samples were used for each group. Asterisk indicates a significant difference compared to the counterpart control group. *p* < 0.05 was considered as the level of significant difference.

The Jak2/STAT5 signaling pathway can be activated by a variety of physiological factors during erythropoiesis, of which the best studied is EPO [12,13]. EPO interacts with EPOR to activate this pathway to drive erythro-megakaryopoiesis in mice [12]. Therefore, we hypothesized that HgCl_2_ might drive erythro-megakaryopoiesis via increased EPO-EPOR signaling in the BM of B10.S mice. To test this, we quantified the expression of EPO production in B10.S mice during HgCl_2_ exposure. Unexpectedly, we found that treatment with 50 μM HgCl_2_ did not impact the expression of EPO protein in serum (Figure 6A) or BM (Figure 6B) in B10.S mice. Consistent with this, treatment with 50 μM HgCl_2_ on B10.S mice did not influence the expression of EPO mRNA in the kidney (Figure 6C), which was the major origin for EPO in vivo [50]. Thus, HgCl_2_ did not impact EPO production in B10.S mice. Next, we measured the expression of EPOR in EMPs in the BM of B10.S mice during HgCl_2_ exposure. Interestingly, treatment with 50 μM HgCl_2_ increased the surface expression of EPOR in EMPs in the BM of B10.S mice (Figure 6D). We further confirmed that HgCl_2_ increased the expression of EPOR in EMPs by measuring the expression of EPOR mRNA in FACS-purified EMPs from the BM of B10.S mice treated with 50 μM HgCl_2_ or the control (Figure 6E). 

To test the function of the EPO-EPOR interaction in the activation of the Jak2/STAT5 signaling pathway, FACS-purified EMPs from the BM of control B10.S mice were treated with EPO protein in vitro, and the pathway was evaluated thereafter. We confirmed that treatment with EPO protein increased the expression of p-Jak2 (Figure 6F) and p-STAT5 (Figure 6G) in FACS-purified EMPs in vitro, indicating that EPO activated the Jak2/STAT5 signaling pathway in EMPs in B10.S mice. As HgCl_2_ increased the expression of EPOR in EMPs, we sought to determine whether EMPs from the BM of B10.S mice treated with 50 μM HgCl_2_ were more sensitive to the EPO protein-induced Jak2/STAT5 signaling pathway. To test this, EMPs from the BM of B10.S mice treated with 50 μM HgCl_2_ or the control were purified, and the FACS-purified EMPs were cultured in vitro thereafter to evaluate their responses to the EPO protein and the involvement of the Jak2/STAT5 signaling pathway. As expected, while EPO treatment increased the expression of p-STAT5 by 1.15 folds in purified EMPs from control B10.S mice, EPO treatment increased the expression of p-STAT5 by 1.29 folds in EMPs from B10.S mice treated with 50 μM HgCl_2_ (Figure 6H), indicating that EMPs in the BM of B10.S mice treated with HgCl_2_ were more sensitive to the EPO-induced activation of the Jak2/STAT5 signaling pathway. Moreover, we confirmed that Jak2 mediated the increased expression of p-STAT5 in EPO-treated EMPs, as the Jak2-specific inhibitor fedratinib significantly suppressed the expression of p-STAT5 in EPO-treated EMPs from the BM of B10.S mice treated with 50 μM HgCl_2_ (Figure 6H). 

While a direct action of HgCl_2_ on EMPs suppressed their differentiation, the activation of the Jak2/STAT5 signaling pathway induced by EPO drove EMP differentiation in B10.S mice treated with 50 μM HgCl_2_, indicating that the EPO-induced activation of this pathway was dominant in erythro-megakaryopoiesis during HgCl_2_ exposure. To test this, FACS-purified EMPs from the BM of control B10.S mice were cultured in vitro in the presence or absence of HgCl_2_ and/or EPO. Although a direct action of HgCl_2_ on EMPs decreased the expression of p-Jak2, EPO significantly increased the expression of p-Jak2 in the presence of HgCl_2_ (Figure 6I), indicating that EPO was dominant in activating the Jak2/STAT5 signaling pathway in B10.S mice during HgCl_2_ exposure. 

Collectively, these experiments suggested that treatment with 50 μM HgCl_2_ increased the expression of EPOR in EMPs to enhance the activation of the Jak2/STAT5 signaling pathway in the BM of B10.S mice. 

### 3.9. HgCl_2_ Does Not Impact Erythro-Megakaryopoiesis in DBA/2 Mice

**Figure 7:**
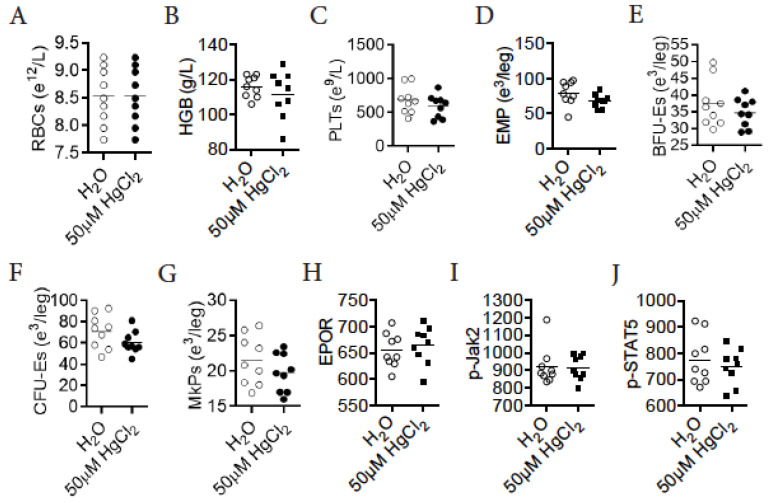
HgCl_2_ does not impact erythro-megakaryopoiesis in DBA/2 mice. DBA/2 mice were treated with 50 μM HgCl_2_ or the control for 4 w, and RBT, erythropoiesis and the Jak2/STAT5 signaling pathway in EMPs were measured thereafter. (**A**) The concentration of RBCs in the blood. (**B**) The concentration of HGB in the blood. (**C**) The concentration of platelets in the blood. (**D**) The number of EMPs in the BM. (**E**) The number of BFU-Es in the BM. (**F**) The number of CFU-Es in the BM. (**G**) The number of MkPs in the BM. (**H**) The expression of EPOR (MFI) in EMPs in the BM. (**I**) The expression of p-Jak2 (MFI) in EMPs in the BM. (**J**) The expression of p-STAT5 (MFI) in EMPs in the BM. Each dot represents one mouse, and a total of nine mice were used for each group. *p* < 0.05 was considered as the level of significant difference.

Finally, we tested whether HgCl_2_ increased erythro-megakaryopoiesis in DBA/2 mice, a strain resistant to Hg-induced autoimmunity [32,33]. Different from B10.S mice, treatment with 50 μM HgCl_2_ did not change the number of mature RBCs, the concentration of HGB, or the number of platelets in the blood of DBA/2 mice (Figure 7A–C). Accordingly, treatment with 50 μM HgCl_2_ did not alter the number of EMPs, BFU-Es, CFU-Es, or MkPs in the BM of DBA/2 mice (Figure 7D–G). Treatment with 50 μM HgCl_2_ did not impact the surface expression of EPOR in EMPs in the BM of DBA/2 mice (Figure 7H). Consistently, treatment with 50 μM HgCl_2_ did not impact the expression of p-Jak2 or p-STAT5 in EMPs in the BM of DBA/2 mice (Figure 7I,J). We therefore concluded that treatment with 50 μM HgCl_2_ did not impact erythro-megakaryopoiesis in DBA/2 mice.

## 4. Discussion

In the present study, we revealed that HgCl_2_ promoted erythro-megakaryopoiesis in the BM of B10.S mice, but not in the BM of DBA/2 mice. 

Erythrocytes are turned over periodically in vivo, depending on the balance between the clearance of senescent erythrocytes in the periphery and newly generated erythrocytes in the BM [16,18]. HgCl_2_ increased the number of progenitors for erythrocytes and megakaryocytes in the BM but did not impact the clearance of RBCs in B10.S mice, indicating that the increased number of mature RBCs and platelets in the blood was due to increased erythro-megakaryopoiesis in B10.S during HgCl_2_ exposure. This was supported by the observation that the number of erythroblasts was also increased in the BM of B10.S mice after treatment with 50 μM HgCl_2_. Notably, while HgCl_2_ drove erythropoiesis in the BM, HgCl_2_ suppressed erythropoiesis in the spleen of B10.S mice, suggesting that an opposite process of erythropoiesis between the BM and periphery occurred during HgCl_2_ exposure, which was in accordance with the observation that mice with increased BM erythropoiesis had reduced peripheral erythropoiesis [16]. 

In the BM, EMPs are able to differentiate into both erythrocytes and megakaryocytes/platelets [7,8,9]. As EMPs are the ancestors for both erythrocyte and megakaryocyte lineages, the increased number of EMPs was sufficient to result in increased erythro-megakaryopoiesis in the BM of B10.S mice after treatment with HgCl_2_. In addition, EMPs from the BM of B10.S mice treated with HgCl_2_ had increased capacity to give rise to more BFU-Es, CFU-Es, and MkPs, indicating that HgCl_2_ also promoted the potential for EMP differentiation. The increased number of EMPs was associated with the increased proliferation of EMPs in the BM of B10.S mice, suggesting that EMPs might self-renew via increased proliferation to some extent during HgCl_2_ exposure. 

Intriguingly, a direct action of Hg^2+^ on FACS-purified EMPs from the BM of B10.S mice actually suppressed their potential for differentiation in vitro. Thus, Hg^2+^ increased erythro-megakaryopoiesis likely through an indirect action on EMPs in the BM of B10.S mice. It should be noted that the suppressive effect of Hg^2+^ on EMP differentiation was tested using in vitro assays, while the Hg^2+^ increase of this differentiation was measured using in vivo assays. 

Previously, we reported that B10.S mice treated with 50 μM HgCl_2_ increased the expression of IFNγ in the BM [19]. In contrast to increased erythro-megakaryopoiesis, IFNγ is actually known to suppress erythropoiesis, which was partially through induction of the PU.1/IRF8 pathway [16]. Thus, the increased erythro-megakaryopoiesis in the BM of B10.S mice after exposure to HgCl_2_ was likely independent of IFNγ signaling. 

EPO is a protein primarily produced by the kidney; during hematopoiesis in the BM, EPO plays an essential role in promoting erythro-megakaryopoiesis, thus leading to the generation of more erythrocytes and megakaryocytes/platelets [50,51]. For instance, EPO drives the proliferation and differentiation of erythrocyte progenitors in the BM of mice [12,50]. Treatment with HgCl_2_ did not impact EPO production, as the expression of EPO mRNA in the kidney was not changed in B10.S mice treated with HgCl_2_. Moreover, the EPO protein concentration in the BM and serum was not impacted by HgCl_2_ exposure in B10.S mice, indicating that the increased amount of EMPs was not caused by increased EPO protein in the BM. Interestingly, HgCl_2_ increased the expression of EPOR in EMPs in the BM of B10.S mice, thus making EMPs more sensitive in response to EPO stimulation. Indeed, some physiological factors do promote erythro-megakaryopoiesis via an increase of EPOR signaling on erythrocyte progenitors, e.g., GM-CSF [52,53]. In terms of the mechanism for differential Hg^2+^ influences in the regulation of EPOR expression in EMPs in the BM of B10.S mice and DBA/2 mice, although further studies are required to address this mechanism, we propose that Hg^2+^ might induce an autoimmune environment to promote certain inflammatory cytokines, which acted on EMPs to increase their expression of EPOR and thereby drove erythro-megakaryopoiesis in the BM of B10.S mice. Hg did not induce autoimmunity in DBA/2 mice, which might account for the observation that Hg did not influence erythro-megakaryopoiesis in DBA/2 mice. 

Regarding the observation that Hg^2+^ suppressed EMP differentiation in vitro while promoting it in vivo in B10.S mice, one explanation was that, although a direct action of Hg on EMPs had a suppressive effect on it, the EMPs in B10.S mice had increased EPOR expression and were thus more sensitive to EPO-induced signaling. Relative to the suppressive effect of Hg^2+^ on EMP differentiation, the enhanced EPO-EPOR signaling in EMPs dominated the increased erythro-megakaryopoiesis in B10.S mice during Hg^2+^ exposure. 

A variety of signaling pathways have been suggested to impact erythro-megakaryopoiesis, of which the Jak2/STAT5 signaling pathway activated by EPO interaction with EPOR is crucial [12,13,15]. Indeed, EPO activated this pathway in EMPs in the BM of B10.S mice. HgCl_2_ promoted the potential for EMP differentiation in the BM of B10.S mice via the induction of this pathway, which was in accordance with the increased expression of EPOR in EMPs. Therefore, it was suggested that treatment with HgCl_2_ activated the pathway by increasing the expression of EPOR in EMPs to promote their potential for differentiation in the BM of B10.S mice.

Our study suggested that the Hg^2+^ increase in erythro-megakaryopoiesis in the BM was related to Hg-induced autoimmunity. It has been well known that Hg-induced autoimmunity is critically dependent on the genetic background of mice, in particular the H-2 haplotypes [25,30]. HgCl_2_ promoted erythro-megakaryopoiesis in B10.S mice, a strain sensitive to Hg-induced autoimmunity, but not in DBA/2 mice, a strain resistant to Hg-induced autoimmunity, indicating that the impact of Hg^2+^ on the development of erythrocytes and megakaryocytes is also likely related to H-2 haplotypes and an autoimmune environment in the BM. In terms of the mechanism for the differential effects of Hg on erythro-megakaryopoiesis in B10.S mice and DBA/2 mice, although it is still unclear, we propose that certain cytokines that were produced in the Hg-induced autoimmune microenvironment in the BM of B10.S mice, but not in DBA/2 mice, induced the increased expression of EPOR in EMPs, thus causing increased activation of the Jak2/STAT5 signaling pathway to drive the differentiation of EMPs. Notably, a previous study reported that exposure to HgCl_2_ for 90 d reduced the number of mature erythrocytes in the blood of rats [6], which in fact does not contradict the observations in our study. Indeed, in addition to genetic background, long-term exposure to Hg was actually able to suppress the autoimmunity of hosts [25]. 

The aberrant accumulation of erythrocytes and platelets may lead to severe clinical symptoms; the most studied is polycythemia vera, which is characterized by the accumulation of morphologically normal erythrocytes, platelets and myeloid cells [54,55]. Clinically, erythrocytosis is the most common and obvious manifestation for polycythemia vera. Notably, while the expression of EPO is not recognized as a good marker for diagnosis, the erythroid progenitors are more proliferative and are hypersensitive to EPO in polycythemia vera patients [55,56], which is similar to the process that EMPs are more responsive to EPO, are more proliferative, and are able to generate more mature erythrocytes in B10.S mice by HgCl_2_ exposure. To date, the etiology of polycythemia vera remains to be defined. To our knowledge, the impacts of environmental factors on polycythemia vera are largely unknown. Thus, as a pilot study, our study may provide a clue for identifying potential environmental risk factors for polycythemia vera in the future. 

Notably, it should be mentioned that previous reports by others showed that Hg^2+^ directly interacted with mature RBCs to impact their redox state and enzyme activation in both humans and animals [4,5,57], which is not discrepant from our results, as we focused on the quantity of RBC generation. Although we did not perform experiments to test the impacts of HgCl_2_ on the function of mature erythrocytes in B10.S mice or DBA/2 mice, we expected the function of mature erythrocytes to be impaired by a direct action of HgCl_2_, probably in both B10.S mice and DBA/2 mice.

Our study may contribute to the current literature on the hematopoietic toxicology of Hg and benefit future risk assessments of Hg in environmental and occupational health. 

The limitations of our study include the absence of both an examination of the effect of HgCl_2_ on an induced anemia mouse model and of the quantification of Hg concentration in the bone.

## 5. Conclusions

**Figure 8:**
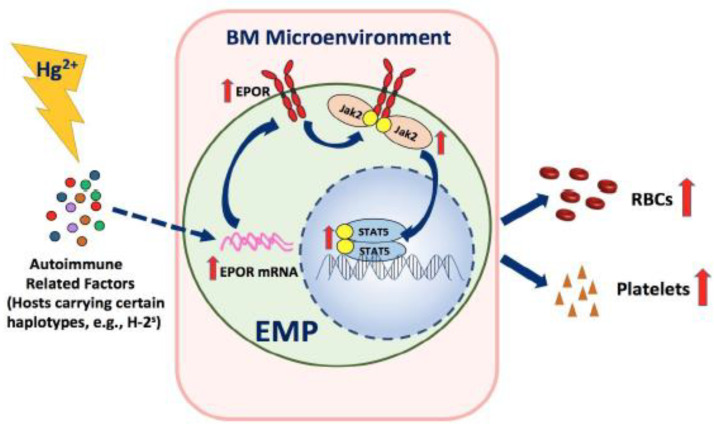
A schematic model for the impact of Hg^2+^ on erythro-megakaryopoiesis in mice. In hosts carrying certain H-2 haplotypes sensitive to Hg-induced autoimmunity (e.g., B10.S mice, H-2^s^), Hg^2+^ induces an autoimmune environment, which leads to the increased expression of EPOR in EMPs and the resultant hypersensitivity of EMPs in response to EPO in the BM. Consequently, the Jak2/STAT5 signaling pathway is overactivated by EPO interaction with EPOR in EMPs, thus resulting in the increased production of RBCs and platelets in the BM. Hg^2+^ does not impact erythro-megakaryopoiesis in mice carrying H-2 haplotypes resistant to Hg-induced autoimmunity (e.g., DBA/2 mice, H-2^d^).

In the present study, as summarized in Figure 8, we have found that HgCl_2_ promoted erythro-megakaryopoiesis via the increased expression of EPOR and the resultant enhanced activation of the Jak2/STAT5 signaling pathway in the BM of B10.S mice; HgCl_2_ did not impact the erythro-megakaryopoiesis in DBA/2 mice.

## Figures and Tables

**Table 1:** Gating strategies and abbreviations for erythro- and megakaryocyte progenitors and erythroblasts.

Cell Type	Abbreviation	Gating Strategy
Erythrocyte-megakaryocyte progenitors	EMPs	Lin^−^c-Kit^hi^Scal-1^−^CD41^−^CD150^+^CD105^−^
Burst-forming unit-erythroid progenitors	BFU-Es	Lin^−^c-Kit^hi^Scal-1^−^CD41^−^CD150^+^CD105^+^
Colony-forming unit-erythroid progenitors	CFU-Es	Lin^−^c-Kit^hi^Scal-1^−^CD41^−^CD150^−^CD105^+^
Megakaryocyte progenitors	MkPs	Lin^−^c-Kit^hi^Scal-1^−^CD41^+^
Erythroblast 1	E1	CD71^+^Ter119^−^
Erythroblast 2	E2	CD71^+^Ter119^+^
Erythroblast 3	E3	CD71^−^Ter119^+^

**Table 2:** Primer information for q-PCR.

Genes	Primers	
	Forward	Reverse
*cdk2*	5′-CTCTCACGGGCATTCCTCTTC-3′	5′-CCCTCTGCATTGATAAGCAGG-3′
*cdk4*	5′-AAGGTCACCCTAGTGTTTGAGC-3′	5′-CCGCTTAGAAACTGACGCATTAG-3′
*epo*	5′-CATCTGCGACAGTCGAGTTCTG-3′	5′-CACAACCCATCGTGACATTTTC-3′
*epor*	5′- GGACCCTCTCATCTTGACGC-3′	5′- CTTGGGATGCCAGGCCAGAT-3′
*β-actin*	5′-GGACTTCGAGCAAGAGATGG-3′	5′-AGCACTGTGTTGGCGTACAG-3′

**Table 3:** Hg concentration (mean ± StD) in the blood and BM of mice.

Treatment		Dose	B10.S		DBA/2	
			Blood (μg/L, ppb)	BM (μg/kg protein, ppb)	Blood (μg/L, ppb)	BM (μg/kg protein, ppb)
H_2_O			ND	ND	ND	ND
HgCl_2_ (μM)		10	26.5 ± 4.3	ND	–	–
		50	242.5 ± 34.2	2500.0 ± 800.0	487.2 ± 65.2	2200.0 ± 600.0

Note: B10.S mice and DBA/2 mice were treated 10 μM or 50 μM HgCl_2_ for 4 week; regular drinking water was used as vehicle control. Total Hg was measured in the blood and BM using atomic fluorescence spectrometry. ND indicates not detectable; – indicates not applicable. A total of 4 to 5 mice were used for each group.

## Data Availability

Not applicable.

## Supplementary Materials

The following are available online at https://www.mdpi.com/article/10.3390/toxics9100252/s1, Figure S1: HgCl_2_ does not impact the clearance of erythrocytes in B10.S mice, Figure S2: A schematic model for erythro-megakaryopoiesis, Figure S3: HgCl_2_ reduces the number of erythroblasts in the spleen of B10.S mice, Figure S4: Schematic models for in vitro assays.

## References

[1] Kim K.H., Kabir E., Jahan S.A. (2016). A review on the distribution of Hg in the environment and its human health impacts. J. Hazard. Mater..

[2] Park J.D., Zheng W. (2012). Human exposure and health effects of inorganic and elemental mercury. J. Prev. Med. Public Health.

[3] Bjorklund G., Dadar M., Mutter J., Aaseth J. (2017). The toxicology of mercury: Current research and emerging trends. Environ. Res..

[4] Ahmad S., Mahmood R. (2019). Mercury chloride toxicity in human erythrocytes: Enhanced generation of ROS and RNS, hemoglobin oxidation, impaired antioxidant power, and inhibition of plasma membrane redox system. Environ. Sci. Pollut. Res. Int..

[5] Temel Y., Taysi M.S. (2019). The Effect of Mercury Chloride and Boric Acid on Rat Erythrocyte Enzymes. Biol. Trace Elem. Res..

[6] Boujbiha M.A., Ben Salah G., Ben Feleh A., Saoudi M., Kamoun H., Bousslema A., Ommezzine A., Said K., Fakhfakh F., El Feki A. (2012). Hematotoxicity and genotoxicity of mercuric chloride following subchronic exposure through drinking water in male rats. Biol. Trace Elem. Res..

[7] Purton L.E., Scadden D.T. (2008). The Hematopoietic Stem Cell Niche.

[8] Wang L.D., Wagers A.J. (2011). Dynamic niches in the origination and differentiation of haematopoietic stem cells. Nat. Rev. Mol. Cell Biol..

[9] Ward C.M., Ravid K. (2020). Matrix Mechanosensation in the Erythroid and Megakaryocytic Lineages. Cells.

[10] Deleschaux C., Moras M., Lefevre S.D., Ostuni M.A. (2020). An Overview of Different Strategies to Recreate the Physiological Environment in Experimental Erythropoiesis. Int. J. Mol. Sci..

[11] Raghuwanshi S., Dahariya S., Musvi S.S., Gutti U., Kandi R., Undi R.B., Sahu I., Gautam D., Paddibhatla I., Gutti R.K. (2019). MicroRNA function in megakaryocytes. Platelets.

[12] Kuhrt D., Wojchowski D.M. (2015). Emerging EPO and EPO receptor regulators and signal transducers. Blood.

[13] Lai X., Nikolov S., Wolkenhauer O., Vera J. (2009). A multi-level model accounting for the effects of JAK2-STAT5 signal modulation in erythropoiesis. Comput. Biol. Chem..

[14] Chang H.C., Huang D.Y., Wu M.S., Chu C.L., Tzeng S.J., Lin W.W. (2017). Spleen tyrosine kinase mediates the actions of EPO and GM-CSF and coordinates with TGF-beta in erythropoiesis. Biochim. Biophys. Acta Mol. Cell Res..

[15] Lappin T.R., Lee F.S. (2019). Update on mutations in the HIF: EPO pathway and their role in erythrocytosis. Blood Rev..

[16] Libregts S.F., Gutiérrez L., de Bruin A.M., Wensveen F.M., Papadopoulos P., van Ijcken W., Özgür Z., Philipsen S., Nolte M.A. (2011). Chronic IFN-gamma production in mice induces anemia by reducing erythrocyte life span and inhibiting erythropoiesis through an IRF-1/PU.1 axis. Blood.

[17] Worley J.R., Parker G.C. (2019). Effects of environmental stressors on stem cells. World J. Stem Cells.

[18] Scharf P., Broering M.F., Oliveira da Rocha G.H., Farsky S.H.P. (2020). Cellular and Molecular Mechanisms of Environmental Pollutants on Hematopoiesis. Int. J. Mol. Sci..

[19] Li Q., Yang Z., Zhang P., Zhao Y., Yu X., Xue P., Shao Y., Li Q., Jia X., Zhang Q. (2018). Mercury impact on hematopoietic stem cells is regulated by IFNgamma-dependent bone marrow-resident macrophages in mice. Toxicol. Lett..

[20] Li Q., Zhang P., Yu X., Zhao Y., Li Q., Zhang Y., Yang Z., Xie Y., Xue P., Sun S. (2017). Lead Transiently Promotes Granulocyte-Macrophage Progenitor Differentiation and Subsequently Suppresses Common Myeloid Progenitor Differentiation. Toxicol. Sci..

[21] Zhang Y., Yu X., Sun S., Li Q., Xie Y., Li Q., Zhao Y., Pei J., Zhang W., Xue P. (2016). Cadmium modulates hematopoietic stem and progenitor cells and skews toward myelopoiesis in mice. Toxicol. Appl. Pharmacol..

[22] Zhao Y., Li Q., Yang Z., Shao Y., Xue P., Qu W., Jia X., Cheng L., He M., He R. (2018). Cadmium Activates Noncanonical Wnt Signaling to Impair Hematopoietic Stem Cell Function in Mice. Toxicol. Sci..

[23] Zhu T., Zhao Y., Zhang P., Shao Y., He J., Xue P., Zheng W., Qu W., Jia X., Zhou Z. (2020). Lead Impairs the Development of Innate Lymphoid Cells by Impeding the Differentiation of Their Progenitors. Toxicol. Sci..

[24] Li Q., Yang Z., Zhao Y., Jia X., Zhou Z., Zhang Y. (2018). Phenotypic and Functional Evaluation of Hematopoietic Stem and Progenitor Cells in Toxicology of Heavy Metals. Curr. Protoc. Toxicol..

[25] Vas J., Monestier M. (2008). Immunology of mercury. Ann. N. Y. Acad. Sci..

[26] Pollard K.M., Lee D.K., Casiano C.A., Bluthner M., Johnston M.M., Tan E.M. (1997). The autoimmunity-inducing xenobiotic mercury interacts with the autoantigen fibrillarin and modifies its molecular and antigenic properties. J. Immunol..

[27] Pollard K.M., Pearson D.L., Bluthner M., Tan E.M. (2000). Proteolytic cleavage of a self-antigen following xenobiotic-induced cell death produces a fragment with novel immunogenic properties. J. Immunol..

[28] Kono D.H., Balomenos D., Pearson D.L., Park M.S., Hildebrandt B., Hultman P., Pollard K.M. (1998). The prototypic Th2 autoimmunity induced by mercury is dependent on IFN-gamma and not Th1/Th2 imbalance. J. Immunol..

[29] Yang Z., Zhao Y., Li Q., Shao Y., Yu E., Cong W., Jia X., Qu W., Cheng L., Xue P. (2019). Developmental exposure to mercury chloride impairs social behavior in male offspring dependent on genetic background and maternal autoimmune environment. Toxicol. Appl. Pharmacol..

[30] Hu H., Moller G., Abedi-Valugerdi M. (1998). Non-responsiveness to mercury-induced autoimmunity in resistant DBA/2 mice is not due to immunosuppression or biased Th1-type response. Scand. J. Immunol..

[31] Hultman P., Bell L.J., Enestrom S., Pollard K.M. (1993). Murine susceptibility to mercury. II. autoantibody profiles and renal immune deposits in hybrid, backcross, and H-2d congenic mice. Clin. Immunol. Immunopathol..

[32] Zhang Y., Bolivar V.J., Lawrence D.A. (2012). Developmental exposure to mercury chloride does not impair social behavior of C57BL/6 x BTBR F(1) mice. J. Immunotoxicol..

[33] Zhang Y., Bolivar V.J., Lawrence D.A. (2013). Maternal exposure to mercury chloride during pregnancy and lactation affects the immunity and social behavior of offspring. Toxicol. Sci..

[34] Zhang Y., Gao D., Bolivar V.J., Lawrence D.A. (2011). Induction of autoimmunity to brain antigens by developmental mercury exposure. Toxicol. Sci..

[35] Bose-O’Reilly S., Bernaudat L., Siebert U., Roider G., Nowak D., Drasch G. (2017). Signs and symptoms of mercury-exposed gold miners. Int. J. Occup. Med. Environ. Health.

[36] Gao Z.-Y., Li M.-M., Wang J., Yan J., Zhou C.-C., Yan C.-H. (2018). Blood mercury concentration, fish consumption and anthropometry in Chinese children: A national study. Environ. Int..

[37] Zhang Y., Jones M., McCabe A., Winslow G.M., Avram D., MacNamara K.C. (2013). MyD88 signaling in CD4 T cells promotes IFN-gamma production and hematopoietic progenitor cell expansion in response to intracellular bacterial infection. J. Immunol..

[38] Zhang Y., Thai V., McCabe A., Jones M., MacNamara K. (2014). Type I interferons promote severe disease in a mouse model of lethal ehrlichiosis. Infect. Immun..

[39] Kippler M., Gyllenhammar I., Glynn A., Levi M., Lignell S., Berglund M. (2020). Total mercury in hair as biomarker for methylmercury exposure among women in central Sweden- a 23 year long temporal trend study. Environ. Pollut..

[40] Li Y., Hu W., Zhao J., Chen Q., Wang W., Li B., Li Y.F. (2019). Selenium decreases methylmercury and increases nutritional elements in rice growing in mercury-contaminated farmland. Ecotoxicol. Environ. Saf..

[41] Dev A., Fang J., Sathyanarayana P., Pradeep A., Emerson C., Wojchowski D.M. (2010). During EPO or anemia challenge, erythroid progenitor cells transit through a selectively expandable proerythroblast pool. Blood.

[42] Yamanegi K., Yamada N., Nakasho K., Nishiura H. (2018). Erythroblast differentiation at spleen in Q137E mutant ribosomal protein S19 gene knock-in C57BL/6J mice. Immunobiology.

[43] Di Giandomenico S., Kermani P., Mollé N., Yabut M.M., Abu-Zeinah G., Stephens T., Messali N., Schreiner R., Brenet F., Rafii S. (2020). Megakaryocyte TGFbeta1 partitions erythropoiesis into immature progenitor/stem cells and maturing precursors. Blood.

[44] Zhang M., Zhu X., Zhang Y., Cai Y., Chen J., Sivaprakasam S., Gurav A., Pi W., Makala L., Wu J. (2015). RCAD/Ufl1, a Ufm1 E3 ligase, is essential for hematopoietic stem cell function and murine hematopoiesis. Cell Death Differ..

[45] Böiers C., Buza-Vidas N., Jensen C.T., Pronk C.J., Kharazi S., Wittmann L., Sitnicka E., Hultquist A., Jacobsen S.E. (2010). Expression and role of FLT3 in regulation of the earliest stage of normal granulocyte-monocyte progenitor development. Blood.

[46] Chang C.-F., Massey J., Osherov A., da Costa L.H.A., Sansing L. (2020). Bexarotene Enhances Macrophage Erythrophagocytosis and Hematoma Clearance in Experimental Intracerebral Hemorrhage. Stroke.

[47] Chohan T.A., Qian H., Pan Y., Chen J.Z. (2015). Cyclin-dependent kinase-2 as a target for cancer therapy: Progress in the development of CDK2 inhibitors as anti-cancer agents. Curr. Med. Chem..

[48] Alvarez-Fernandez M., Malumbres M. (2020). Mechanisms of Sensitivity and Resistance to CDK4/6 Inhibition. Cancer Cell.

[49] Suresh S., Rajvanshi P.K., Noguchi C.T. (2019). The Many Facets of Erythropoietin Physiologic and Metabolic Response. Front. Physiol..

[50] Hacein-Bey-Abina S., Estienne M., Bessoles S., Echchakir H., Pederzoli-Ribeil M., Chiron A., Aldaz-Carroll L., Leducq V., Zhang Y., Souyri M. (2020). Erythropoietin is a major regulator of thrombopoiesis in thrombopoietin-dependent and -independent contexts. Exp. Hematol..

[51] Blake T.J., Jenkins B.J., D’Andrea R.J., Gonda T.J. (2002). Functional cross-talk between cytokine receptors revealed by activating mutations in the extracellular domain of the beta-subunit of the GM-CSF receptor. J. Leukoc. Biol..

[52] Fisher J.W. (2003). Erythropoietin: Physiology and pharmacology update. Exp. Biol. Med. (Maywood).

[53] Hultman P., Bell L.J., Enestrom S., Pollard K.M. (1992). Murine susceptibility to mercury. I. Autoantibody profiles and systemic immune deposits in inbred, congenic, and intra-H-2 recombinant strains. Clin. Immunol. Immunopathol..

[54] McMullin M.F. (2016). Investigation and Management of Erythrocytosis. Curr. Hematol. Malig. Rep..

[55] Spivak J.L. (2002). Polycythemia vera: Myths, mechanisms, and management. Blood.

[56] Davila-Gonzalez D., Barrios-Ruiz A., Fountain E., Cheng L., Masarova L., Verstovsek S., Rojas-Hernandez C.M. (2020). Diagnostic Performance of Erythropoietin Levels in Polycythemia Vera: Experience at a Comprehensive Cancer Center. Clin. Lymphoma Myeloma Leuk..

[57] Hassanin M., Kerek E., Chiu M., Anikovskiy M., Prenner E.J. (2016). Binding Affinity of Inorganic Mercury and Cadmium to Biomimetic Erythrocyte Membranes. J. Phys. Chem. B.

