# Supplementary material for: Mercury Chloride Impacts on the Development of Erythrocytes and Megakaryocytes in Mice
Source: Toxics. 2021 Oct 7;9(10):252. doi: 10.3390/toxics9100252 (PMC8537753; doi:10.3390/toxics9100252)
Supplement: Supplementary file 1 [file toxics-09-00252-s001.zip › toxics-1405805-supplementary.pdf]

toxics

MDPI

Article

Supplementary Materials: Mercury Chloride Impacts on the Development of Erythrocytes and Megakaryocytes in Mice

Jinyi He, Yifan Zhao, Tingting Zhu, Peng Xue, Weiwei Zheng, Ye Yao, Weidong Qu, Xiaodong Jia, Rongzhu Lu, Miao He and Yubin Zhang

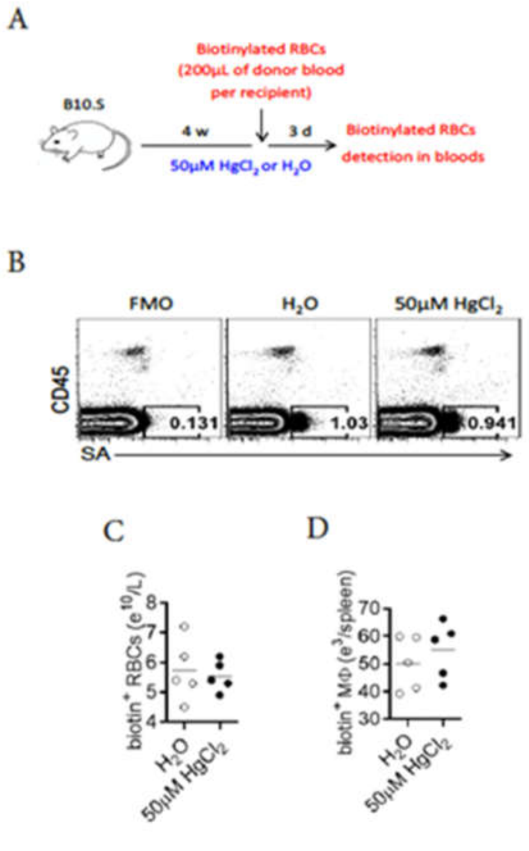

Figure S1. $HgCl_2$ does not impact the clearance of erythrocytes in B10.S mice**.** Biotinylated RBCs from regular B10.S mice were intravenously injected into control or 50 μM $HgCl_2$-treated B10.S mice, and the clearance of the biotinylated (biotin⁺) RBCs was measured thereafter. **A:** A schematic model for RBC clearance detection. **B:** Representative flow plots for the donor (biotinylated) RBCs in the peripheral blood of recipients. **C:** Absolute number of donor RBCs as indicated in **A** and **B**. **D**: Quantification of biotin⁺ MΦ in the spleen of recipients as indicated in **A**. Each dot represents one mouse, and a total of 5 mice were used for each group. $p < 0.05$ was considered as the level of significant difference.

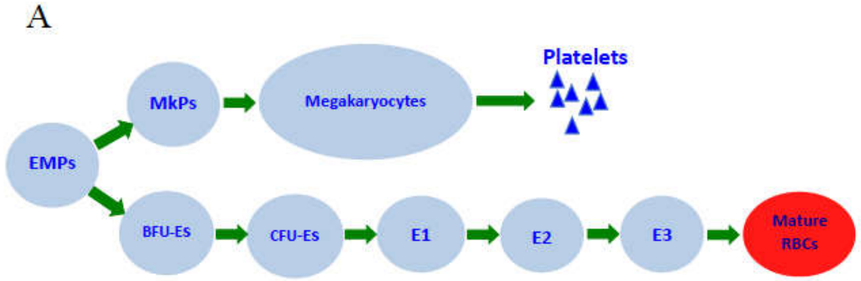

Figure S2. A schematic model for erythro-megakaryopoiesis.

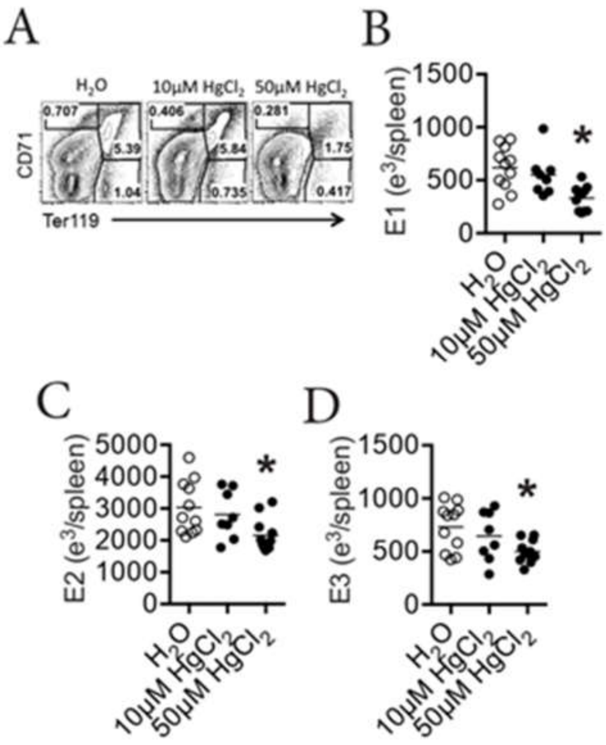

Figure S3. $HgCl_2$ reduces the number of erythroblasts in the spleen of B10.S mice**.** B10.S mice were treated with 10 μM or 50 μM $HgCl_2$ for 4 w and the number of erythroblasts (E1, E2 and E3) in the spleen was performed thereafter. A: Representative flow plots for E1, E2 and E3 in the spleen. B: Quantification of E1 in the spleen as indicated in A. C: Quantification of E2 in the spleen as indicated in A. D: Quantification of E3 in the spleen as indicated in A. Each dot represents one mouse, and a total of 8 to 11 mice were used for each group. Asterisk indicates a significant difference compared to the counterpart control group. $p < 0.05$ was considered as the level of significant difference.

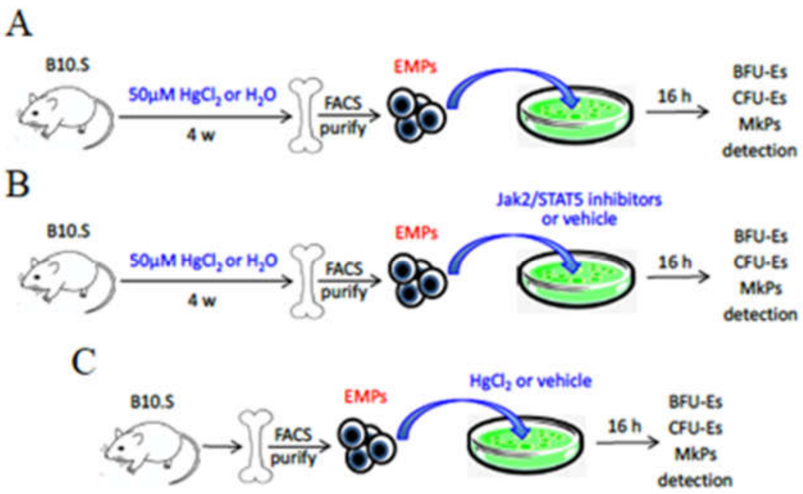

Figure S4. Schematic models for in vitro assays. **A**: A schematic model for EMP differentiation assay in vitro. **B**: A schematic model for evaluating the role of the Jak2/STAT5 signaling pathway in EMP differentiation during $HgCl_2$ exposure. **C**: A schematic model for EMP differentiation in the presence or absence of $HgCl_2$ in vitro.
